# Impact of Haemodialysis on Insulin Kinetics of Acute Kidney Injury Patients in Critical Care

**DOI:** 10.1007/s40846-015-0015-x

**Published:** 2015-02-03

**Authors:** Ummu K. Jamaludin, Paul D. Docherty, J. Geoffrey Chase, Geoffrey M. Shaw

**Affiliations:** 1 Faculty of Mechanical Engineering, Universiti Malaysia Pahang, 26600 Pekan, Pahang Malaysia; 2 Department of Mechanical Engineering, Centre of Bioengineering, University of Canterbury, Private Bag 4800, Christchurch, 8140 New Zealand; 3 Department of Intensive Care Christchurch School of Medicine and Health Science, PO Box 4345, Christchurch, 8140 New Zealand

**Keywords:** Insulin sensitivity, Tight glycaemic control, Haemodialysis, Intensive care unit, Acute kidney injury

## Abstract

Critically ill patients are occasionally associated with an abrupt decline in renal function secondary to their primary diagnosis. The effect and impact of haemodialysis (HD) on insulin kinetics and endogenous insulin secretion in critically ill patients remains unclear. This study investigates the insulin kinetics of patients with severe acute kidney injury (AKI) who required HD treatment and glycaemic control (GC). Evidence shows that tight GC benefits the onset and progression of renal involvement in precocious phases of diabetic nephropathy for type 2 diabetes. The main objective of GC is to reduce hyperglycaemia while determining insulin sensitivity. Insulin sensitivity (*S*_*I*_) is defined as the body response to the effects of insulin by lowering blood glucose levels. Particularly, this study used *S*_*I*_ to track changes in insulin levels during HD therapy. Model-based insulin sensitivity profiles were identified for 51 critically ill patients with severe AKI on specialized relative insulin nutrition titration GC during intervals on HD (OFF/ON) and after HD (ON/OFF). The metabolic effects of HD were observed through changes in *S*_*I*_ over the ON/OFF and OFF/ON transitions. Changes in model-based *S*_*I*_ at the OFF/ON and ON/OFF transitions indicate changes in endogenous insulin secretion and/or changes in effective insulin clearance. Patients exhibited a median reduction of −29 % (interquartile range (IQR): [−58, 6 %], *p* = 0.02) in measured *S*_*I*_ after the OFF/ON dialysis transition, and a median increase of +9 % (IQR −15 to 28 %, *p* = 0.7) after the ON/OFF transition. Almost 90 % of patients exhibited decreased *S*_*I*_ at the OFF/ON transition, and 55 % exhibited increased *S*_*I*_ at the ON/OFF transition. Results indicate that HD commencement has a significant effect on insulin pharmacokinetics at a cohort and per-patient level. These changes in metabolic behaviour are most likely caused by changes in insulin clearance or/and endogenous insulin secretion.

## Introduction

Acute kidney injury (AKI) is a common complication among critically ill patients, especially for elderly patients with diabetes [1]. Approximately 36 % of critically ill patients are diagnosed with AKI [1–3] with a significant proportion progressing to severe AKI (Stage 3) [2], requiring weekly haemodialysis (HD) [4]. Several epidemiological studies have shown an increase in morbidity and mortality following the development of severe AKI [3, 5–7].

The increasing incidence of critically ill patients with severe AKI may be explained by several factors, including a rising incidence of sepsis [3, 4], major surgery (especially cardiothoracic), nephrotoxic medications, and chronic medical conditions [5]. With both uraemia and HD treatment, glycaemic control (GC) can be complicated [6] as GC affects insulin secretion, insulin clearance, gluconeogenesis [7], and peripheral tissue sensitivity of insulin [8]. Many studies have claimed that HD treatment is necessary to treat severe AKI patients by removing waste and toxins [9]. Other clinical studies have shown that HD treatment cleared plasma insulin through increased absorption [10–12] through the dialyzer membrane, lowering insulin concentration. Also, abnormalities in insulin secretion have important pathophysiological implications in the genesis of AKI, which is responsible for the progressive reduction in insulin requirement of diabetic patients who develop AKI [13]. However, the effect of renal failure on insulin kinetics in critically ill patients is still unclear due to the lack of pharmacokinetic studies on insulin secretion and clearance related to HD treatment. These unknown effects might have the potential to complicate metabolic management and the treatment itself.

In particular, insulin resistance is common in many severe AKI patients [4, 14, 15]. Hence, these patients are at risk of developing hyperglycaemia [6] with its associated negative outcomes [16, 17]. The mechanism of glucose intolerance in severe AKI patients is ambiguous [18]. DeFronzo et al. [21] and Mak [20] showed that insulin resistance among severe AKI patients improved during a 10-week course of HD treatment. However, the net effect of HD treatment on glycaemic regulation and insulin sensitivity (*S*_*I*_) in a critically ill cohort is unknown.

This study uses dense clinical data and a model-based analysis to investigate changes in a clinically validated model-based *S*_*I*_ metric at HD transitions in a cohort of critically ill patients. It was hypothesized that the observed *S*_*I*_ would decrease during HD due to enhanced insulin clearance compared to the model and would be recaptured again when HD is stopped. These changes in model-based *S*_*I*_ would thus offer a unique observation of insulin kinetics and action in this population of critically ill patients with severe AKI that would better inform metabolic care.

## Materials and Methods

### Patient Cohort

Retrospective blood glucose (*G*) measurements, nutrition administration rates (*P*), and insulin delivery (*U*_*X*_) data used in this study were obtained from the Specialized Relative Insulin Nutrition Titration (SPRINT) pilot study of 371 critically ill patients who required GC [21]. 51 of the 371 patients had severe AKI treated with HD. The exogenous insulin and nutrition given to these patients were optimized to maximise blood glucose time in the range of 4.0–7.0 mmol L^−1^, minimising hyperglycaemia, via patient-specific nutrition and insulin administration [12].

The 51 severe AKI patients were treated with HD with a polysulfone (PS) dialyzer membrane (APS-15SA: Asahi Medical Co., Ltd, Tokyo). This PS dialyzer membrane has been reported to affect plasma insulin clearance during HD treatment [22, 23]. Patients were subjected to HD three times weekly (in a fasting state) for a minimum of 4 h in the Christchurch Hospital Intensive Care Unit (ICU).

Study inclusion from 51 severe AKI patients required a minimum of 5 h of patient data before dialysis, followed by at least 6 h of dialysis, and then at least 5 h after dialysis. The clinical details of this cohort are summarized in Table 1. The APACHE III diagnosis for these patients can be divided into 5 main groups: Sepsis, Cardiovascular, Trauma, Respiratory, and Diabetes. Full details on SPRINT can be obtained elsewhere [21].

**Table 1:** SPRINT cohort baseline variables (N = 51)

	Median	[IQR]
Age (years)	65	46–73
% male	76 %	
APACHE II score	24	19–30

APACHE III Diagnosis	Number of patients	%
Trauma	9	18
Cardiovascular	13	25
Sepsis	20	39
Respiratory	7	14
^a^Diabetes	2	4

^a^Patients diagnosed with type 1 diabetes mellitus (T1DM)

Data are expressed as median values [IQR] (APACHE = Acute Physiology And Chronic Health Evaluation)

### Limitation of Study

The study is mainly focused on the (intermittent) HD treatment as one of many treatments in managing severe AKI. Continuous renal replacement therapy (life-supporting treatments) is commonly instituted in critical care when severe AKI is diagnosed. Continuous renal replacement therapy [9] includes:

- HD
- peritoneal dialysis
- hemofiltration
- renal transplantation

All of these treatments are regarded as life-extending treatments to support renal function. With dense clinical data obtained from the SPRINT glycaemic protocol [21], it is suggested for most critically ill patients to be treated with HD in order to avoid other metabolic complications such as sepsis and trauma [15]. Furthermore, the effects of continuous renal replacement inter-therapies (as mentioned above) can be discovered with a larger cohort with different AKI by observing the changes in insulin sensitivity that affects insulin kinetics.

### Identification of Model-Based *S*_*I*_

Model-based *S*_*I*_ was identified hourly by fitting *G* measurements with estimated endogenous insulin secretion using the ICING (Intensive Control Insulin-Nutrition-Glucose) model [24] with modification. An integral-based method [25] and clinical data were used to identify a patient-specific stepwise *S*_*I*_ profile with a 1 h resolution. The model nomenclatures are given in Table 2. It is mathematically defined as:

1 $$\mathop G\limits^{ \bullet } (t) = - p_{G} G(t) - S_{I} G(t)\frac{Q(t)}{{1 + \alpha_{G} Q(t)}} + \frac{P(t) + EGP - CNS}{{V_{G} }}$$

2 $$\mathop Q\limits^{ \bullet } (t) = n_{I} (I(t) - Q(t)) - n_{C} \frac{Q(t)}{{1 + \alpha_{G} Q(t)}}$$

3 $$\mathop I\limits^{ \bullet } (t) = - n_{K} I(t) - n_{L} \frac{I(t)}{{1 + \alpha_{I} I(t)}} - n_{I} (I(t) - Q(t)) + \frac{{u_{ex} }}{{V_{I} }} + (1 - x_{L} )\frac{{u_{en} (G)}}{{V_{I} }}$$

4 $$P_{ 1} \left( t \right) = - d_{ 1} P_{ 1} + D\left( t \right)$$

5 $$P_{ 2} \left( t \right) = - { \hbox{min} } \left( {d_{ 2} P_{ 2} , P_{ \hbox{max} } } \right) + d_{ 1} P_{ 1}$$

6 $$P\left( t \right) = { \hbox{min} } \left( {d_{ 2} P_{ 2} , P_{ \hbox{max} } } \right) + PN\left( t \right)$$

7 $$u_{{en}} (G) = \left\{ {\begin{array}{*{20}l} {u_{{\min }} ,} \hfill & {u_{{\min }} > k_{1} G(t) + k{}_{2}} \hfill \\ {k_{1} G(t) + k_{2} ,} \hfill & {u_{{\min }} \le k_{1} G(t) + k_{2} \le u_{{\max }} } \hfill \\ {u_{{\max }} ,} \hfill & {u_{{\max }} < k_{1} G(t) + k_{2} } \hfill \\ \end{array} } \right.$$

**Table 2:** Nomenclature of ICING-2 Model

Parameters	Description			Unit
*G*	Blood glucose level			mmol L^−1^
*Q*	Interstitial insulin level			mU L^−1^
*I*	Plasma insulin level			mU L^−1^
*P*_*1*_	Stomach glucose content			mmol
*P*_*2*_	Gut glucose content			mmol
*P*	Rate of glucose appearance in plasma			mmol min^−1^
*u*_*en*_	Endogenous insulin secretion rate			mU min^−1^
Parameters and kinetic values of ICING-2 model based on diabetic status				
*EGP*	Endogenous glucose production rate	1.16		mmol min^−1^
*CNS*	Central nervous system glucose uptake	0.3		mmol min^−1^
*p*_*G*_	Patient endogenous glucose removal	0.006		min^−1^
*S*_*I*_	Insulin sensitivity			L mU^−1^ min^−1^
*α*_*G*_	Saturation parameter of insulin-mediated glucose removal	0.0154		L mU^−1^
*V*_*G*_	Plasma glucose distribution volume	13.3		L
*n*_*I*_	Plasma-interstitium insulin diffusion rate	0.006		min^−1^
*n*_*C*_	Receptor-bound insulin degradation	0.006		min^−1^
*n*_*K*_	Renal insulin clearance	0.0542		min^−1^
*n*_*L*_	Hepatic insulin clearance	0.1578		min^−1^
*α*_*I*_	Saturation parameter for hepatic insulin clearance	0.0017		L mU^−1^
*V*_*I*_	Insulin distribution volume	4.0		L
*x*_*L*_	First pass hepatic clearance	0.67		
*d*_*1*_	Rate of glucose transport through the enteral route into the bloodstream	0.0347		min^−1^
*d*_*2*_		0.0069		min^−1^
*P*_*max*_	Maximal gut glucose flux	6.11		mmol min^−1^
*u*_*max*_	Maximum pancreatic secretion rate	266.7		mU min^−1^
*u*_*min*_	Minimum pancreatic secretion rate	16.7		mU min^−1^
*k*_*1*_	Pancreatic insulin secretion glucose-sensitivity	*NGT	14.9	mU L mmol^−1^ min^−1^
		*T2DM	4.9	
		*T1DM	0.0	
*k*_*2*_	Pancreatic insulin secretion offset	*NGT	−49.9	mU·min^−1^
		*T2DM	−27.4	
		*T1DM	16.7	
Exogenous input variables of ICING-2 model				
*u*_*ex*_	Intravenous insulin input rate			mU min^−1^
*D*	Oral glucose input rate from enteral nutrition			mmol min^−1^
*PN*	Intravenous glucose input rate from parenteral nutrition			mmol min^−1^

* NGT = normal glucose tolerance, T1DM = type 1 diabetes mellitus, T2DM = type 2 diabetes mellitus

Model estimation of endogenous insulin secretion (*u*_*en*_(*G*)) is in the range of 16.7–266.7 mU min^−1^ as a function of glycaemic level (*G*) [26]. This overall metabolic model has been clinically validated with a median prediction error of less than 5 % [27]. The model has been used in several clinical GC trials and insulin sensitivity tests [28, 29].

### Hypothesis

Specifically, constant *n*_*K*_ is used in model ICING describes in Eqs. (1)–(6). While, *u*_*en*_ model in Eq. (7) is dependent on *G* values. Therefore, the unmodeled changes due to HD or any other effect [30] are reflected in the model-based *S*_*I*_. Two dialysis transitions, OFF/ON and ON/OFF, are examined in this study.

#### OFF/ON Transition

The HD PS membrane is known to absorb plasma insulin during dialysis treatment [23]. Therefore, it was hypothesized that after the OFF/ON transition, *S*_*I*_ will decrease given a model assumption of a fixed *u*_*en*_ which dependent on *G* and a constant *n*_*K*_.

#### ON/OFF Transition

By using the same dialyzer membrane with the assumptions of a fixed *u*_*en*_ (dependable on *G*) and a constant *n*_*K*_, it was hypothesized that *S*_*I*_ will increase as the plasma insulin level recovers to higher levels after HD treatment ends.

Thus, changes in *S*_*I*_ due to HD might be caused either by:

- changes in *u*_*en*_ due to HD treatment [14, 23]
- changes in the effective insulin clearance (*n*_*K*_ in the model) [31, 32]

However, only changes and the net effect of *S*_*I*_ after both transitions were tracked so the separate effects could not be delineated. In particular, a rising of *u*_*en*_ that is based on the assumption of a fixed *u*_*en*_ (dependable on *G*) leads to an increase in the observed *S*_*I*_, while rising *n*_*K*_ leads to an apparent reduction in *S*_*I*_. If *S*_*I*_ is decreasing, it means that the effect of insulin clearance increases outweighs the effect of *u*_*en*_ increases.

### Calculations and Statistical Analysis

Numerical calculations and parameter identification were performed using MATLAB (The MathWorks Inc., Natick, MA). The proportional difference in *S*_*I*_ (Δ*S*_*I*_) was calculated as:

8 $$\Delta S_{I} = 2\frac{{S_{I(after)} - S_{I(before)} }}{{\left( {S_{I(before)} + S_{I(after)} } \right)}}$$

Blood glucose changes, Δ*G*, were calculated in a manner similar to that for Δ*S*_*I*_ to assess any changes in glycaemia that could affect results.

This analysis uses a 2 h moving average to reduce the effect of measurement error and the influence of transient effects. *S*_*I*_ profiles are identified over periods starting at 3 h before dialysis commencement until 4 h after dialysis ends. This range ensures full settling of patient responses after transitions. The patients’ blood glucose and insulin sensitivity at both OFF/ON and ON/OFF dialysis transitions are illustrated on distribution and Bland–Altman plots.

Non-parametric Wilcoxon rank sum tests were used to assess ∆*S*_*I*_ and ∆G over the cohort at each transition.

## Results

**Fig. 1:**
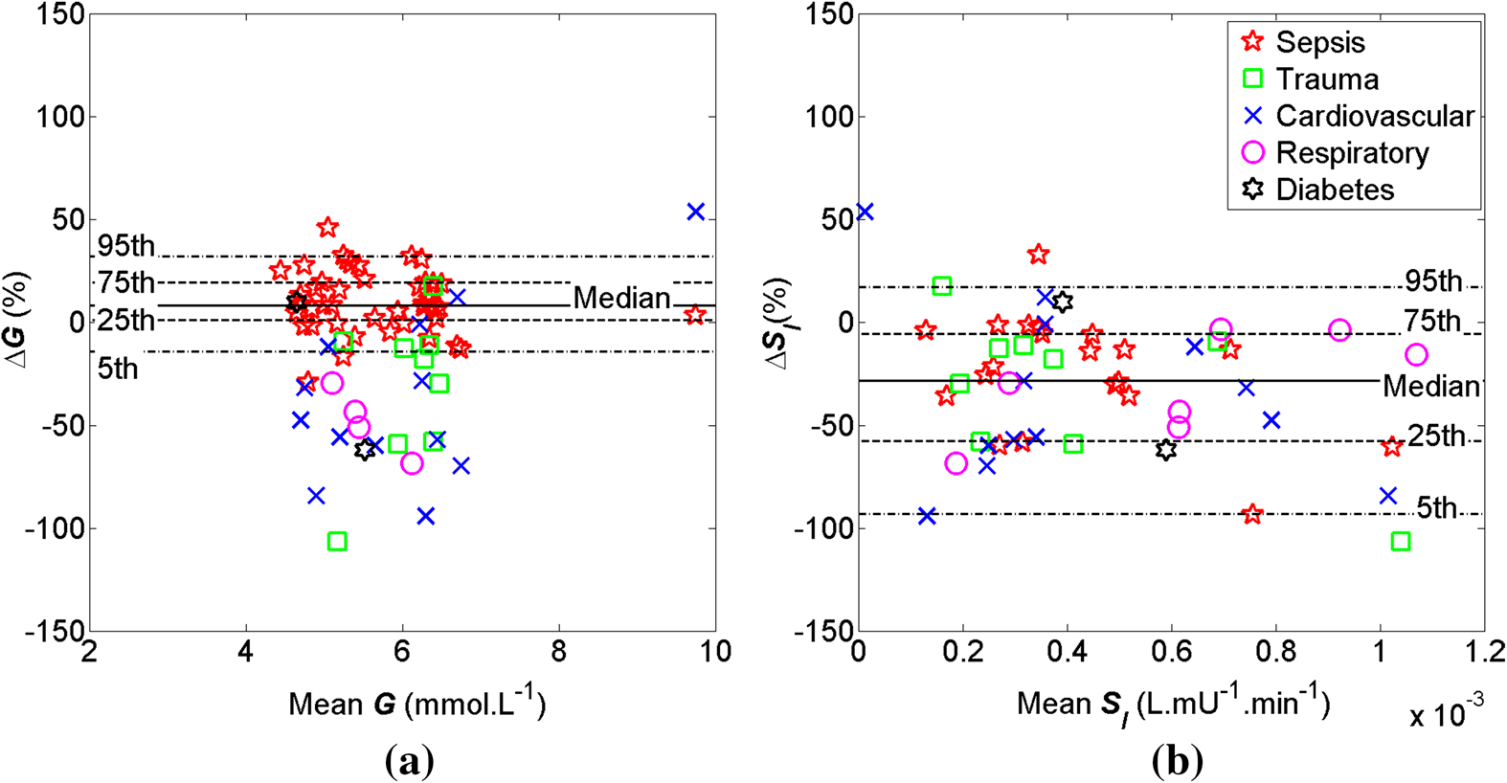
Bland–Altman plots of **a** Δ*G* and **b** Δ*S*_*I*_ over the OFF/ON dialysis transition between t = −1 and 2 for severe AKI patients (N = 51). Median Δ*G* = 8 % and median Δ*S*_*I*_ = −29 %

**Fig. 2:**
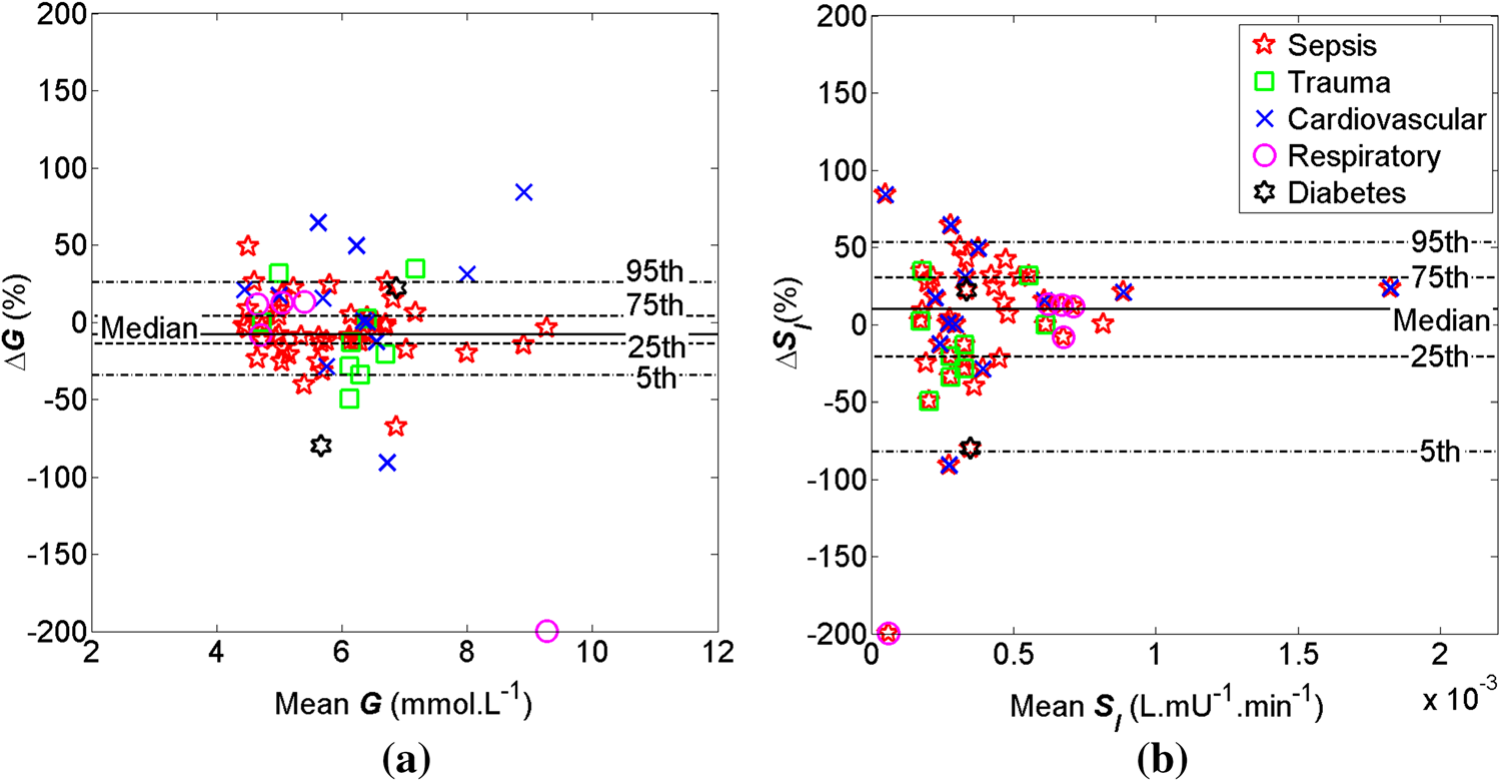
Bland–Altman plots of **a** Δ*G* and **b** Δ*S*_*I*_ over the ON/OFF dialysis transition between t = −1 and 2 for severe AKI patients (N = 51). Median Δ*G* = −8 % and median Δ*S*_*I*_ = 10 %

Figures 1 and 2 show Bland–Altman plots of ∆*G* and ∆*S*_*I*_ over the OFF/ON and ON/OFF dialysis transitions. ∆*G* and ∆*S*_*I*_ at the ON/OFF transition are unbiased overall, but *S*_*I*_ is biased (median ∆*S*_*I*_ = −29 %, *p* = 0.02) over the OFF/ON dialysis transition (Fig. 1b).

At the OFF/ON dialysis transition, the *G* distribution is effectively maintained within 4–7 mmol L^−1^ except for two patients with sepsis and cardiovascular diagnoses.

**Fig. 3:**
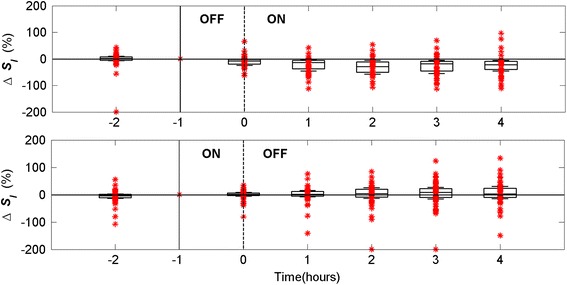
Patient distribution for dialysis period of 6 h at OFF/ON (**a**) and ON/OFF (**b**) dialysis transitions from t = −2 to t = 4 h

Figure 3 shows Δ*S*_*I*_ over 6 h at the OFF/ON and ON/OFF dialysis transitions. Patients diagnosed with pancreatitis, diabetes, and other metabolic dysfunctions showed larger variance in Δ*S*_*I*_ (>−150 %). However, the trend at the OFF/ON transition in Fig. 3a (and Fig. 1) is much clearer.

Table 3 summarises Δ*S*_*I*_ over the OFF/ON and ON/OFF transitions. *S*_*I*_ decreased after the OFF/ON dialysis transition until t = 2 h, where it settled with median ∆*S*_*I*_ = −29 % (interquartile range (IQR): [−58,6] %, *p* = 0.02). There were a comparatively low number of confounders, indicating a relatively strong effect. Median Δ*S*_*I*_ increased by 9 % for the ON/OFF transition (Table 3), (IQR: [−15,28] %; *p* = 0.7) at t = 3 h after the ON/OFF transition. The number of confounders is significantly higher for the ON/OFF transition and the *p* values indicate that the hypothesized effect cannot be confirmed at this transition. *G* remains effectively constant at both transitions (Figs. 1, 2). However, changes in *S*_*I*_ outcomes were not significant (*p* > 0.05) even 4 h after the ON/OFF transition.

**Table 3:** Results for OFF/ON and ON/OFF dialysis transitions of 6 h with inverted *S*_*I*_ confounders (t = −2 to t = 4, N = 51)

Time t (hr)	OFF/ON (N = 51), expect Δ*S*_*I*_ < 0						ON/OFF (N = 51), expect Δ*S*_*I*_ > 0					
	Q_I_ (%)	Q_2_ (%)	Q_3_ (%)	*p* value	Δ*S*_*I*_ > 0 % (confounders)		Q_1_ (%)	Q_2_ (%)	Q_3_ (%)	*p* value	Δ*S*_*I*_ < 0 % (confounders)	
−2	−7	1	10	0.9	31	61	−13	−4	3	0.7	32	63
−1	0	0	0	1	0	0	0	0	0	1	0	0
0	−24	−7	−1	0.3	10	20	−5	1	8	0.9	24	47
1	−45	−14	−2	0.05	7	14	−7	2	15	0.9	23	45
2	−58	−29	−6	0.02	5	10	−13	4	26	0.8	21	41
3	−55	−19	−5	0.03	9	18	−15	9	28	0.7	23	45
4	−46	−22	−5	0.03	6	12	−15	3	31	0.5	24	47

The results show Δ*S*_*I*_ quartiles and number of confounders with Δ*S*_*I*_ in direction opposite to median trend hypothesized. Q_2_ = median = 50 % percentiles result. * *p* values measured using Wilcoxon rank sum tests, Q_1_ = 25 % percentile, Q_3_ = 75 % percentile

An extended dialysis interval (>10 h) of Δ*S*_*I*_ for both OFF/ON and ON/OFF dialysis transitions across the N = 26 subjects with sufficient data is shown in Table 4. *S*_*I*_ decreased during the OFF/ON dialysis interval until t = 8 h, where it settled to a median reduction of −25 % (IQR: [−10, −51] %; *p* = 0.04). There were only 2 confounders (Δ*S*_*I*_ > 0) from the 26 patients at t = 8 h. However, while the ON/OFF transition results improved relative to the hypothesized effect, the results were still insignificant (*p* > 0.07).

**Table 4:** Extended results for OFF/ON and ON/OFF dialysis transitions of >10 h with inverted *S*_*I*_ confounders (t = −2 to t = 10, N = 26)

Time t (hr)	OFF/ON (N = 26), expect Δ*S*_*I*_ < 0						ON/OFF (N = 26), expect Δ*S*_*I*_ > 0					
	Q_I_ (%)	Q_2_ (%)	Q_3_ (%)	*p* value	Δ*S*_*I*_ > 0 % (confounders)		Q_1_ (%)	Q_2_ (%)	Q_3_ (%)	*p* value	Δ*S*_*I*_ < 0 % (confounders)	
−2	−4	3	13	0.9	16	62	−15	−6	1	0.7	19	73
−1	0	0	0	1	0	0	0	0	0	1	0	0
0	−25	−10	−2	0.4	4	15	−5	3	9	0.8	10	28
1	−44	−23	−4	0.1	3	12	−7	8	17	0.8	11	42
2	−56	−30	−6	0.09	2	8	−8	13	32	0.6	9	35
3	−55	−19	−2	0.09	6	23	−4	18	42	0.3	9	35
4	−53	−24	−4	0.1	4	15	−13	14	36	0.3	10	28
5	−53	−29	−8	0.08	4	15	−12	22	44	0.2	10	28
6	−44	−25	−8	0.1	4	15	−6	23	47	0.1	8	31
7	−40	−21	−9	0.07	3	12	−5	18	50	0.07	7	27
8	−51	−25	−10	0.04	2	8	−5	19	39	0.07	7	27
9	−47	−18	−7	0.04	1	4	−6	22	38	0.07	8	31
10	−45	−12	−3	0.09	5	19	−4	22	41	0.08	8	31

The results show Δ*S*_*I*_ quartiles and number of confounders with Δ*S*_*I*_ in direction opposite to median trend hypothesized. Q_2_ = median = 50 % percentile result. * p values measured using Wilcoxon rank sum tests, Q_1_ = 25 % percentile, Q_3_ = 75 % percentile

## Discussion

This study investigated the effect of dialysis on renal insulin clearance, endogenous insulin secretion, and effective plasma insulin through a clinically validated model-based Δ*S*_*I*_ metric at both OFF/ON and ON/OFF dialysis transitions. Significant insulin sensitivity changes were observed at the OFF/ON dialysis transition (*p* = 0.02). This analysis indicates that model-based *S*_*I*_ decreased over the initial 4 h after HD started and that the changes occurred as rapidly as 2 h. This result implies that dialysis significantly affects plasma insulin levels via changes in renal insulin clearance and/or endogenous insulin secretion, compared to baseline model assumptions.

Glucose intolerance among critically ill patients with severe AKI occurs with significant inhibition of insulin secretion and a state of peripheral insulin resistance [14, 33] on top of insulin resistance from the critical illness [34]. It has also been reported that in patients with severe AKI, insulin resistance occurred even though glomerular filtration rate (GFR) values were still within the normal range [35]. The effect of insulin resistance can be exacerbated by impairment of the role of insulin in maintaining the hepatic glucose balance [19]. Specifically, an inability of insulin to stimulate hepatic glucose uptake with decreasing *S*_*I*_ has been observed in severe AKI patients [36]. Thus, understanding the pharmacokinetics of insulin during dialysis is clinically important.

Plasma insulin is reduced by enhanced insulin clearance due to the PS dialyzer membrane [22, 23] used during HD treatment in this study. It is suggested that the most significant reduction in plasma insulin during HD treatment is through absorption of insulin across the PS membrane [23], where the equilibrium amount of insulin absorbed was greatest in positively charged membranes [31]. A significant uptake and degradation of insulin may occur when renal insulin clearance significantly exceeds the glomerular filtration rate [6], as would occur in HD treatment. This enhanced insulin clearance rate and accumulation of dialyzable uraemic toxins can cause inhibition of insulin degradation and can be sufficiently normalized by HD treatment [20].

Plasma insulin levels also depend on endogenous insulin secretion. Physiologically, *u*_*en*_ is determined by the glycaemic level and the ability of β cells to respond to blood glucose level and its changes. However, it was suggested that an increase in endogenous insulin secretion may occur in response to HD treatment with a PS membrane dialyzer due to reductions in plasma insulin [10, 22]. In particular, a PS membrane can reduce plasma insulin significantly in HD [10, 23]. Thus, *S*_*I*_ is also expected to decrease with an unmodeled increase in *u*_*en*_ during the initial period of HD treatment to maintain the *G* level.

The model-based Δ*S*_*I*_ at the ON/OFF dialysis transition in this study was insignificant (*p* > 0.05). It is assumed that acute intravenous (i.v.) administration of 1,25-dihydroxyvitamin D_3_ (1,25(OH)_2_D_3_) given to severe AKI patients during HD may increase insulin secretion and reverse glucose intolerance [20]. An improvement in glucose metabolism has been observed in some studies via lower mean glucose during dialysis and a more rapid disappearance rate of glucose in the immediate post-dialysis period [37]. In general, glucose metabolism and renal function are expected to increase gradually after post-dialysis when toxic substances that are suspected of hindering renal function have been extracted. Long-term (4.9 weeks) HD treatment has been shown to normalize insulin sensitivity and result in a marked improvement in glucose metabolism [11], but this might not completely normalize glucose utilization [19]. Overall, it is impossible to delineate the effects that contribute to changes in *S*_*I*_ in this study, due to model identifiability issues [16] and the side effects of other diagnosed critical illnesses apart from severe AKI [18]. Over longer intervals, as in Table 4, inter-patient or intra-patient variation may further obscure the observation of the effect itself [28].

Thus, a substantial change in *S*_*I*_ at the OFF/ON dialysis transition indicates a strong and fast process of the cleaning and clearing of toxic substances from blood, improving effective *S*_*I*_ due to either decreased *u*_*en*_ or increased *n*_*K*_ clearance. However, at the ON/OFF dialysis transition, the recovery process to regulate and normalize blood is a lot slower physiologically. Hence, the model-based *S*_*I*_ after dialysis may be expected to remain unchanged, as observed here, even for extended periods after HD treatment.

The model-based *S*_*I*_ is an indication of overall glucose metabolism of critically ill patients and does not necessarily reflect the precise cellular physiology of peripheral insulin sensitivity. The model-based Δ*S*_*I*_ at a cohort level used in this study are unlikely to be caused by actual variance in true peripheral *S*_*I*_ at a cellular level. In particular, there is no apparent stimulus induced by HD to directly affect *S*_*I*_. Thus, Δ*S*_*I*_ reflects changes in renal clearance or/and endogenous insulin secretion, which in turn result in changes in the model-based *S*_*I*_ calculated based on fixed assumptions for these values.

In particular, the ICING model prediction of *u*_*en*_ is made in terms of blood glucose level in the absence of direct measurement of C-peptide. Hence, the effect of dialysis on *u*_*en*_ cannot be defined patient-specifically by the model without added data that was not available in this study. Alternatively, it has been reported that endogenous insulin secretion is also affected by exogenous insulin [38]. As plasma insulin levels are suspected to decrease during dialysis, it is suspected that endogenous insulin secretion would increase at a cohort level. Thus, an increase in the model-based *S*_*I*_ over time should be observed at the OFF/ON transition, contrary to the observations here. Hence, *u*_*en*_ dependence on blood glucose level would confound the observed effect and can be discounted as a contributor to the shifts in the model-based *S*_*I*_ in this study. Thus, it is most likely that effective renal clearance increases during HD and decreases after HD treatment.

Effective *u*_*en*_ identification cannot be undertaken with only glucose data [16, 39]. Thus, a direct measurement of C-peptide should be included for direct quantification of the effects contributing to the glycaemia of severe AKI patients. The results of this study could be used to confirm these results in order to power a further study that segregates these potential contributing effects.

All critically ill patients with severe AKI in this study were undergoing SPRINT tight glycaemic control (TGC), where the minimal changes in *G* illustrated that SPRINT was successful in controlling glycaemia during these transitions. A consistent trend of Δ*G* below the 5th percentile was observed for patients with cardiovascular, respiratory, and trauma compared to the patient diagnosed with sepsis, as shown in Fig. 1a. It portrays sepsis complications due to severe infections and multiple organ dysfunctions [40], which were not the main focus in this study. Thus, glycaemic levels and the tightness of this protocol are used to ensure that the analysis results are not biased by variations in glucose levels which can affect stress response and thus *S*_*I*_ levels. SPRINT clinical results were also compared with the simulation results to validate and minimize the prediction errors of the protocol. Although there were two diagnosed diabetics, almost no bias in Δ*G* was observed. Thus, the confounding factor plays no role.

Overall, this investigation suggests that the most likely contributor to the observed changes in *S*_*I*_ was the HD insulin clearance, which was modeled by the renal insulin clearance term. The effect of HD on plasma insulin and the mechanism of insulin clearance among critically ill patients with severe AKI were shown in this study to be a contributor to overall effective *S*_*I*_, which determines the glycaemic level, all else being equal. However, further in-depth studies must be undertaken to measure the specific effects of HD in different AKI stages. A prospective cohort and clinical studies with direct insulin and C-peptide assays on this cohort may lead to a better understanding of insulin kinetics during HD treatment. A broad comparison from a different cohort of varied HD duration with mixed levels of insulin will also clarify the effects of Δ*S*_*I*_, revealing further details in the underlying contributors of specific insulin resistance.

## Conclusion

The distinct change in model-based insulin sensitivity during HD treatment was a significant and observable aspect of critically ill patient physiology. The findings were consistent with the presence of effects of HD treatment in a majority of severe AKI patients from other studies. Clinically, the effect of the main contributors (*n*_*K*_ and *u*_*en*_) to effective insulin sensitivity changes during HD from a baseline model or clinical assumptions suitable for other patients should also be considered in GC. However, the precise pharmaco-kinetics/dynamics driving this change remain ambiguous. These results justify a larger cohort investigation with specific measurement of insulin secretion and renal clearance to differentiate these impacts.
